# TRPC3 contributes to cyclophosphamide-induced cystitis progression by enhancing bladder fibrosis through activation of the TGF-β/smad pathway

**DOI:** 10.3389/fphar.2025.1565156

**Published:** 2025-04-09

**Authors:** Ruixiang Luo, Wenshuang Li, Junlong Huang, Zheng Liu, Chi Zhang, Honglu Ding, Jialiang Chen

**Affiliations:** Department of Urology, The Third Affiliated Hospital of Sun Yat-sen University, Guangzhou, China

**Keywords:** interstitial cystitis, bladder pain syndrome, bladder fibrosis, TRPC3, TGF-β

## Abstract

**Background:**

Bladder pain syndrome/Interstitial cystitis (BPS/IC) is a chronic urological disorder affecting 2.7%–6.5% of the population. The condition is characterized by significant bladder-related pain, with approximately 50% of IC/BPS patients exhibiting bladder fibrosis. Transient receptor potential cation channel subfamily C member 3 (TRPC3), a protein linked to fibrosis in heart and kidneys, emerged as a potential therapeutic target for this condition.

**Methods:**

Using a cyclophosphamide-induced cystitis rat model, we employed RNA sequencing for transcriptional profiling, Western blot for protein quantification, and Masson staining for fibrotic assessment. Cellular-specific TRPC3 expression patterns were elucidated through single-cell transcriptomic analysis. TRPC3 inhibition was implemented via intraperitoneal administration of Pyrazole 3. The study assessed mechanical pain sensitivity and bladder function through von Frey testing and cystometry.

**Results:**

Significant findings revealed TRPC3 RNA and protein expression was markedly upregulated in cystitis rats. TRPC3 inhibition substantially improved mechanical pain sensitivity and reduced micturition frequency. TRPC3 is predominantly expressed in fibroblasts and fibrosis-related pathways are upregulated in cystitis rats. The increased fibrosis markers and collagen fiber deposition are both reversed by TRPC3 inhibition. And the TGF-β/Smad signaling pathway was notably activated and subsequently downregulated with TRPC3 inhibition.

**Conclusion:**

TRPC3 activation contributes significantly to bladder fibrosis in IC/BPS. Inhibiting TRPC3 ameliorates symptoms by modulating TGF-β/Smad pathway, suggesting it as a promising therapeutic target for managing this challenging condition with limited current treatment options.

## Introduction

Interstitial cystitis/bladder pain syndrome (IC/BPS) is characterized by bladder pain or pelvic discomfort related to bladder filling, usually accompanied by lower urinary tract symptoms such as urinary frequency and urgency (Clemens et al., 2022a). Although IC/BPS is not a malignant condition, it significantly impairs quality of life, particularly in women. Currently available treatments are very limited, with most only achieving short-term relief. For IC/BPS patients with decades-long medical histories, clinical benefits are extremely minimal. Moreover, the incidence of IC/BPS is increasing annually (2.7–6.5%) (Berry et al., 2011), and its medical costs are more than twice that of patients of the same age group. Therefore, exploring the key pathogenic mechanisms of IC/BPS is of critical importance for elucidating the essential nature of IC/BPS development and identifying potential new therapeutic targets. The precise mechanisms underlying IC/BPS remain unclear, with hypotheses including urothelial barrier dysfunction, immune system abnormalities, and bladder nerve hypersensitivity. A preliminary clinical investigation with limited sample size indicates that bladder fibrosis is crucial in the progression of IC/BPS, with severe and moderate fibrosis more commonly observed in non-Hunner-type IC (NHIC) compared to Hunner-type IC (HIC) and non-IC cases. Patients with severe fibrosis exhibit significantly higher urinary frequency and reduced bladder capacity compared to those with moderate or mild fibrosis (Kim et al., 2017). Additionally, detrusor fibrosis can lead to ineffective standard urological treatments, necessitating more intensive therapy for IC/BPS patients (Richter et al., 2010). *In vivo* studies have shown that exposure of human epithelial SV-HUC-1 cells to TNF-α results in increased vimentin and collagen expression, promoting fibrosis (Jin et al., 2021). Nonetheless, the mechanisms driving bladder fibrosis in IC/BPS are still not well understood.

Transient receptor potential cation channel subfamily C member 3 (TRPC3) is a cation channel that regulates calcium ion influx in cells and is involved in various physiological processes, including cell signaling and ion homeostasis (Cole and Becker, 2023). Fibroblasts, which produce collagen, are key players in tissue scarring and excessive collagen deposition. In fibrotic diseases such as renal and atrial fibrosis, TRPC3 has been implicated in promoting fibroblast proliferation, differentiation, and activation by regulating Ca^2+^ influx (Gwanyanya and Mubagwa, 2022; Yan et al., 2022). These findings suggest that TRPC3 may play a role in fibrosis progression by stimulating collagen production through fibroblast activation. However, the involvement of TRPC3 in bladder fibrosis associated with IC/BPS remains unexplored.

Bladder fibrosis often represents a late-stage pathological change in IC/BPS and is a major factor contributing to refractory cases that require more intensive treatment. Unfortunately, current treatment options for improving bladder fibrosis in IC/BPS patients are limited. Given the evidence, we hypothesize that TRPC3 plays a role in the development of bladder fibrosis in IC/BPS through its effects on fibroblasts, and that inhibiting TRPC3 expression could potentially improve this condition. Therefore, we conducted this study to explore the role of TRPC3 in bladder fibrosis associated with IC/BPS and to evaluate it as a novel therapeutic target.

## Materials and methods

### RNA-seq analysis

RNA sequencing was employed for comprehensive transcriptional profiling. Total RNA was extracted and purified using TRIzol reagent (Invitrogen, United States) following the manufacturer’s instructions. The RNA amount and purity of each sample was quantified using NanoDrop ND-1000 spectrophotometer (NanoDrop, United States). The RNA integrity was assessed using a Bioanalyzer 2,100 (Agilent, United States), with an RNA Integrity Number (RIN) greater than 7.0, and further confirmed by denaturing agarose gel electrophoresis. Poly(A) RNA was purified from 1 μg of total RNA using Dynabeads Oligo (dT)25-61005 (Thermo Fisher, United States) with two rounds of purification. The purified poly(A) RNA was then fragmented into small pieces using the Magnesium RNA Fragmentation Module (NEB, United States) at 94°C for 5–7 min. The cleaved RNA fragments were reverse-transcribed to create cDNA using SuperScript™ II Reverse Transcriptase (Invitrogen, United States), and U-labeled second-strand DNA was synthesized using *E. coli* DNA polymerase I (NEB, United States), RNase H (NEB, United States), and dUTP Solution (Thermo Fisher, United States). An A-base was added to the blunt ends of each strand, and indexed adapters containing a T-base overhang were ligated to the fragments. Selection was performed using AMPureXP beads. After treatment with the heat-labile UDG enzyme (NEB, United States), the ligated products were amplified by PCR using the following conditions: initial denaturation at 95°C for 3 min; 8 cycles of denaturation at 98°C for 15 s, annealing at 60°C for 15 s, and extension at 72°C for 30 s; and a final extension at 72°C for 5 min. The average insert size for the final cDNA library was 300 ± 50 bp. Finally, 2 × 150bp paired-end sequencing (PE150) was performed on an illumina Novaseq™ 6,000 system (LC-Bio Technology CO., China) following the vendor’s recommended protocol.

The fastp software (https://github.com/OpenGene/fastp) was used to remove reads containing adaptor contamination, low quality bases, and undetermined bases, using default parameters. Sequence quality was further verified with fastp. HISAT2 (https://ccb.jhu.edu/software/hisat2) was employed to map the reads to the *Homo sapiens* GRCh38 reference genome. The mapped reads for each sample were assembled using StringTie (https://ccb.jhu.edu/software/stringtie) with default parameters. Subsequently, all transcriptomes from all samples were merged to reconstruct a comprehensive transcriptome using gffcompare (https://github.com/gpertea/gffcompare/). After generating the final transcriptome, StringTie was utilized to estimate the expression levels of all transcripts. Specifically, StringTie was employed to quantify mRNA expression levels by calculating FPKM (FPKM = [total_exon_fragments/mapped_reads (millions) × exon_length (kB)]), which is the standard normalization method for paired-end RNA-seq data in major databases (e.g., TCGA and GEO). Differentially expressed mRNAs were identified using the R package DESeq2, with a threshold of fold change >1.5 or <0.67 and a significance level of adjusted p-value <0.05 based on Wald test.

### Identification of differentially expressed genes and gene set enrichment analyses

Differential expression analysis of CYP-induced cystitis rats (n = 3) and normal rats (n = 3) were performed by Novogene Inc., using DESeq2 R package (v1.38.3). The p-values were calculated using the negative binomial distribution and adjusted for multiple testing using the Benjamini–Hochberg method to control the false discovery rate (FDR). The clusterProfiler (v4.6.2) software was used for the gene set enrichment analysis (GSEA), pathways with adjusted p-values of less than 0.05 were considered significantly enriched among the two groups.

### Sources and processing of single-cell sequencing data

This study utilized the single-cell RNA sequencing dataset for interstitial cystitis (GSE175526) obtained from the Gene Expression Omnibus (GEO) database. The data analysis encompassed the following key steps: First, the raw gene expression matrix was processed using the Seurat R package (v4.4.0). Low-quality and dead cells were excluded based on pre-established criteria, which included a minimum of 200 detected genes and a maximum mitochondrial UMI percentage of 15%. Outliers were also removed to enhance the robustness and reliability of downstream analyses. Subsequently, batch effects were corrected using the Harmony algorithm, ensuring harmonization of gene expression across samples. Dimensionality reduction and visualization of cell clusters were achieved using the t-SNE. Finally, cell populations were annotated based on the expression of canonical marker genes, leading to the identification of seven distinct cell subpopulations.

### Animals and treatment

Sprague Dawley (SD) rats (200–250g, 6–8 weeks old) were obtained from the Laboratory Animal Center of Sun Yat-sen University. All animal procedures were approved by the Institutional Animal Care and Use Committee of Sun Yat-sen University (Approval No. SYSU-IACUC-2020-000,412). All Animal experiments all comply with ARRIVE ß(Animal Research: Reporting of *In Vivo* Experiments). The rats were housed continuously in a specific pathogen-free environment. An IC/BPS rat model was established using cyclophosphamide (CYP) (Sigma-Aldrich, United States) as previously described (Chen et al., 2021). Briefly, CYP (50 mg/kg) was administered via intraperitoneal injection every 3 days for a total of three doses. The IC/BPS animal model was validated in our previous study (Chen et al., 2019). A total of 100 animals were included in this study. Of these, 12 were randomly assigned to Control and CYP groups (n = 6 per group), with 3 animals from each group randomly selected for RNA-Seq analysis and the remaining 3 for Western blot qualification of TRPC3 expression. The other 88 animals were randomly distributed among four experimental groups: Control, Pyr3, CYP, and CYP + Pyr3 (n = 22 per group). Within each of these groups, 14 animals were randomly designated for assessment of mechanical allodynia and cystometry, 4 for Masson staining of bladder tissue, and the remaining 4 for Western blot qualification of fibrosis markers and TGF-β/Smad signaling expression.

A selective TRPC3 inhibitor, Pyrazole 3 (Pyr3) (MedChemExpress, United States), was used to investigate the role of TRPC3 in IC/BPS. The Control group consisted of normal rats receiving intraperitoneal saline injections. The Pyr3 group was treated with intraperitoneal injections of Pyr3 (0.1 mg/kg (Kim et al., 2011; Wang et al., 2016) or 1 mg/kg). Both the CYP group and the CYP + Pyr3 group received intraperitoneal injections of CYP to establish the IC/BPS animal model. In the CYP + Pyr3 group, Pyr3 (0.1 mg/kg or 1 mg/kg) was administered intraperitoneally 30 min before each CYP injection and continued for 3 consecutive days following the establishment of the cystitis model, for a total of 6 doses. The Pyr3 group received Pyr3 in the same manner. The CYP group was injected with an equivalent volume of PBS. The time points for CYP and Pyr3 treatment are shown in the top schematic in Figure 2A.

### Assessment of mechanical allodynia

We measured the lower abdominal mechanical threshold as a proxy for evaluating bladder pain in CYP-induced cystitis model, as direct assessment of bladder pain is challenging (Bon et al., 2003). Von-Frey filaments were employed to objectively quantify lower abdominal mechanical thresholds using methodologies previously established in our laboratory (Chen et al., 2019; Chen et al., 2020; Chen et al., 2021). Briefly, the up-down method (Chaplan et al., 1994) and a series of von-Frey filaments (rated at 0.4, 0.6, 1, 2, 4, 6, 8, 15 g) were used to measure evoked pain in the lower abdomen, an area associated with referred pain from vesical pathologies. The 2-g stimulus was applied initially. Each stimulus involved a 6–8-s application of the von Frey filament to the lower abdominal region, with at least 5-minute intervals between applications. After a negative or positive response was observed, a stronger or weaker filament was applied, respectively. A positive response was defined as licking or scratching the stimulated site or flinching or moving away from the stimulus. Mechanical allodynia was assessed every 3 days throughout the experiment.

### Cystometry

Cystometry was conducted following the methods we previously described (Xie et al., 2018; Zhang et al., 2022). Briefly, after the rat was restrained, the bladder was emptied using manual abdominal pressure. A PE-50 catheter was then inserted retrograde into the bladder and connected to a pressure transducer (BL-420F, Taimeng Technology, China) in line with an infusion pump. Sterile normal saline was infused at a rate of 6 mL/h, and intravesical pressure was continuously recorded using BL New Century 2.1 software (Taimeng Technology, China). Once the voiding cycles stabilized (typically after three to four cycles), an additional 30 min of data were recorded for quantitative analysis.

### Western blot analysis

Western blot analysis was conducted to quantify TRPC3 expression, fibrosis-associated markers, and key proteins within the TGF-β/Smad signaling pathway critical to fibrotic progression. Proteins were extracted from bladder tissue using RIPA lysis buffer (Beyotime, China) supplemented with proteinase and phosphatase inhibitors. Protein concentration was determined using a BCA Protein Assay Kit (CWBIO, China). The proteins were separated on a 10% sodium dodecyl sulfate-polyacrylamide gel and transferred to 0.45 μm polyvinylidene fluoride membranes. The membranes were blocked with 5% bovine serum albumin and then incubated with primary antibodies against TRPC3 (Cat#ACC-016, 1:1,000; Alomone Labs, Isreal), Vimentin (Vim, Cat#ab8978, 1:1,000; Abcam, United Kingdom), Collagen I (Col-I, Cat#ab270993, 1:1,000; Abcam, United Kingdom), Collagen III (Col-III, Cat#ab7778, 1:1,000; Abcam, United Kingdom), Transforming growth factor beta (TGF-β, Cat#ab215715, 1:1,000; Abcam, United Kingdom), phospho-Smad2 (*p*-Smad2, Cat#ab188334, 1:1,000; Abcam, United States), phospho-Smad3 (*p*-Smad3, Cat#ab52903, 1:1,000; Abcam, United State) and GAPDH (Cat#2118S, 1:1,000; CST, United States). The membranes were then incubated with Goat Anti-Rabbit IgG (H + L) HRP-conjugated secondary antibody (Cat# S0001, 1:5,000; Affinity, China). Protein bands were visualized using an Enhanced Chemiluminescent Kit (New Cell and Molecular Biotech Co., Ltd., China) on the Tanon 5200 C E Chemi-Image System (Tanon, China). Band intensities were analyzed using ImageJ software (National Institutes of Health, United States), and relative protein expression was normalized to GAPDH.

### Masson staining

Rat bladders were excised via a longitudinal abdominal incision, then fixed in 10% neutral-buffered formalin. The tissue was subsequently dehydrated and embedded in paraffin. Paraffin-embedded tissues were sectioned into 5-μm thickness and subjected to Masson’s trichrome (Sigma-Aldrich, United States) staining according to the manufacturer’s instructions. To assess the results of Masson’s trichrome staining, five randomly selected fields from each bladder section were examined using light-microscopy (Leica Microsystems, Germany) and a computer-assisted morphometric analyzer (Leica Microsystems, Germany). The extent of fibrosis in bladder tissue was quantified by calculating the ratio of collagen area to total tissue area. Collagen area quantification was conducted using an image analysis system (ImageJ, United States).

### Statistical analysis

Statistical analyses were conducted using IBM SPSS Statistics 23.0 (IBM Corporation, United States). Data are presented as mean ± standard error of the mean (SEM). The statistical significance between two groups was assessed using Student’s t-test. One-way analysis of variance (ANOVA) followed by Tukey’s *post hoc* test was used for comparisons of more than three groups. For the mechanical withdrawal threshold results, data were analyzed using a repeated-measures two-way ANOVA followed by a Tukey’s *post hoc* test. The Shapiro-Wilk test was applied to confirm the normality of the data before performing ANOVA. A P-value of <0.05 was considered statistically significant.

## Results

### TRPC3: a crucial gene in CYP-Induced cystitis rats

**FIGURE 1:**
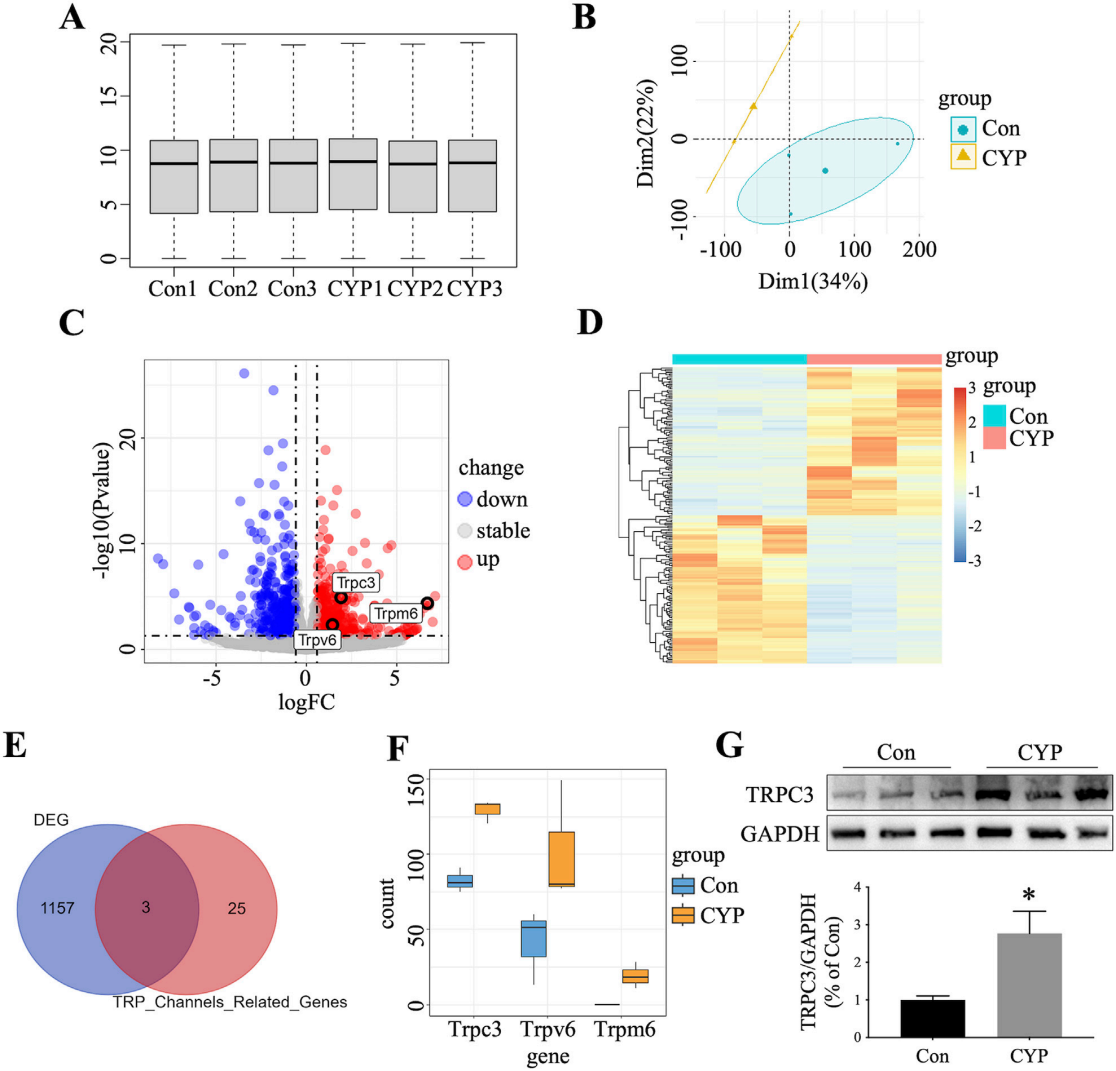
Identification of TRPC3 as A Crucial Gene in CYP-Induced Cystitis Rats. **(A)** Box plot illustrating gene expression data from CYP-induced cystitis rats and control. **(B)** PCA distinguishing the two experimental groups. **(C)** Volcano plot depicting DEGs between the groups. **(D)** Heatmap presenting the top 100 up-regulated and top 100 down-regulated DEGs. **(E)** Venn diagram highlighting the overlap between DEGs and TRP-related genes. **(F)** Expression levels of intersecting genes in the two groups. **(G)** Western blot confirmed the overexpression of TRPC3 in the bladders of CYP-induced cystitis rats. n = 3 per group. The statistical significance between the two groups were assessed using Student's t-test: *P < 0.05 vs Con group.

To identify genes involved in CYP-induced cystitis rats, we conducted bulk RNA-seq analysis on bladder tissue from both CYP-induced cystitis rats (n = 3) and normal rats (n = 3). Various visualizations were created to interpret the data, including a box plot (Figure 1A), principal component analysis (PCA) map (Figure 1B), volcano plot (Figure 1C), and heat map (Figure 1D). As illustrated in Figure 1E, our analysis identified 1,160 differentially expressed genes between the two groups. We then intersected these genes with members of the TRP (transient receptor potential) ion channel family (Zhang et al., 2023), pinpointing three key genes: TRPC3, TRPV6, and TRPM6. Of these, TRPC3 emerged as the most highly upregulated gene in CYP-induced cystitis rats compared to normal rats (Figure 1F). Western blot studies confirmed a substantial increase in TRPC3 expression in the bladders of CYP-induced cystitis rats (Figure 1G), corroborating the findings from our bioinformatics analysis.

### Inhibition of TRPC3 with Pyr3 alleviates bladder pain in CYP-Induced cystitis rats

**FIGURE 2:**
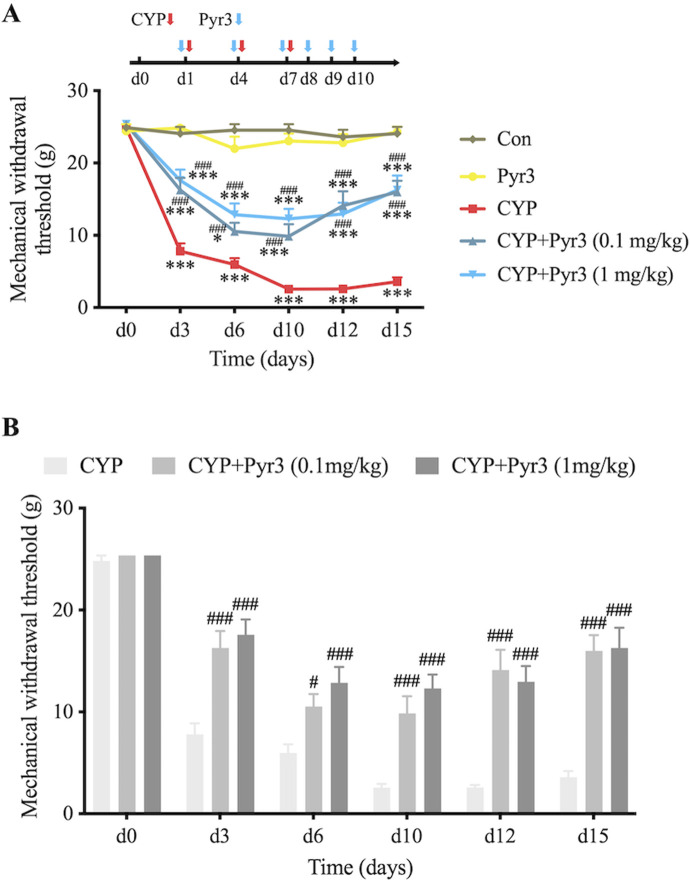
Inhibition of TRPC3 with Pyr3 Alleviates Bladder Pain in CYP-Induced Cystitis Rats. **(A)** Mechanical withdrawal threshold significantly decreased after the initial CYP injection in CYP-induced cystitis rats. Treatment with Pyr3 at both 0.1 mg/kg and 1 mg/kg markedly elevated mechanical withdrawal thresholds at all assessed time points. (B) No significant differences were observed between the two Pyr3-treated groups. n = 14 per group. Statistical significance was determined using two-way ANOVA followed by the Tukey post hoc test: *P < 0.05 and ***P < 0.001 vs Con group, ^#^ *P* < 0.05 and ^###^ *P* < 0.001 vs CYP group.

To investigate the role of TRPC3 in bladder fibrosis, we administered Pyr3, a selective TRPC3 inhibitor, to CYP-induced cystitis rats. Two doses of Pyr3 (0.1 mg/kg and 1 mg/kg) were tested to assess potential dose-dependent effects. Both doses significantly alleviated bladder pain in CYP-induced cystitis rats (Figure 2A), with Pyr3 treatment notably increasing the mechanical withdrawal threshold at all time points, including the day after the initial CYP injection. No significant difference was observed between the two Pyr3-treated groups, suggesting that higher doses do not enhance the treatment effect (Figure 2B). At the final assessment, 5 days after the last Pyr3 injection, the mechanical withdrawal threshold remained significantly lower in both Pyr3-treated groups compared to controls. Additionally, Pyr3 treatment had a minimal impact on normal rats, showing only a slight reduction in the mechanical withdrawal threshold compared to controls.

### Inhibition of TRPC3 with Pyr3 reversed bladder overactivity in CYP-Induced cystitis rats

**FIGURE 3:**
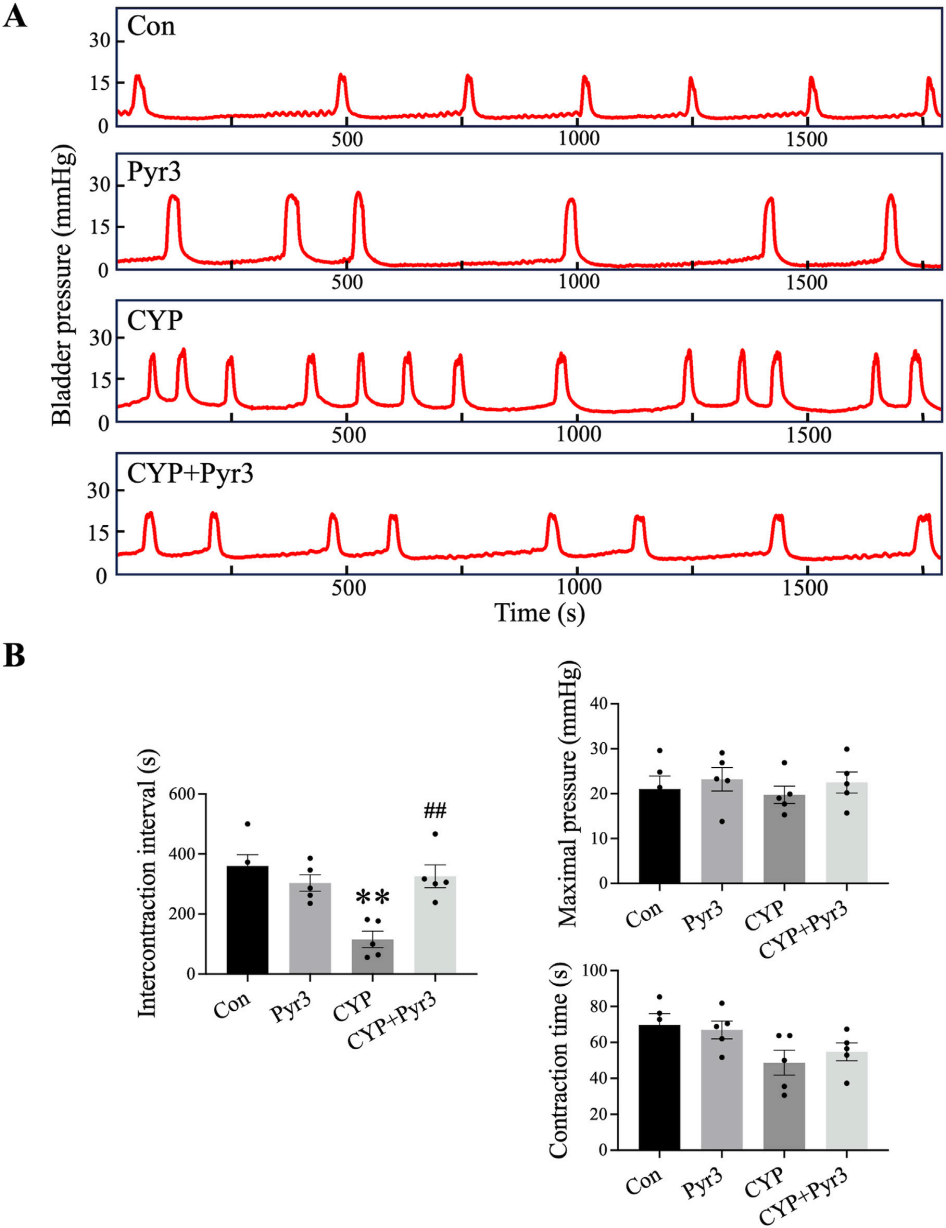
Inhibition of TRPC3 with Pyr3 Ameliorated Bladder Overactivity in CYP-Induced Cystitis Rats. **(A)** Representative bladder pressure traces from each experimental group. **(B)** CYP-induced cystitis rats displayed bladder overactivity, characterized by a significantly shorter intercontractile interval (ICI) compared to controls. Pyr3 treatment effectively restored the ICI to levels observed in the control group. No significant differences were observed in bladder contraction pressure or contraction time across groups. n = 5 per group. Statistical significance was determined using one-way ANOVA followed by the Tukey post hoc test: **P < 0.01 vs Con group, ^##^ *P* < 0.01 vs CYP group.

Considering that the therapeutic effects of Pyr3 at doses of 0.1 mg/kg and 1 mg/kg on bladder pain in CYP-induced cystitis rats are comparable, we used only the 0.1 mg/kg dose of Pyr3 in subsequent experiments. On day 12 after the first CYP injection, also day 2 after the last Pyr3 injection, cystometry was performed to measure the micturition reflex in different animal groups. Figure 3A displays bladder pressure traces from the four groups, showing that CYP-induced cystitis rats exhibited bladder overactivity. Figure 3B illustrates a significantly shorter intercontractile interval (ICI) in the CYP group compared to the control group. Pyr3 treatment effectively reversed the ICI reduction caused by CYP-induced cystitis. However, no significant differences were observed in bladder contraction pressure or contraction time among the four groups. These findings suggest that TRPC3 inhibition with Pyr3 effectively reverses bladder overactivity in CYP-induced cystitis rats.

### TRPC3 is primarily expressed in fibroblasts within bladder tissue, and fibrosis-related pathways are upregulated in CYP-Induced cystitis rats

**FIGURE 4:**
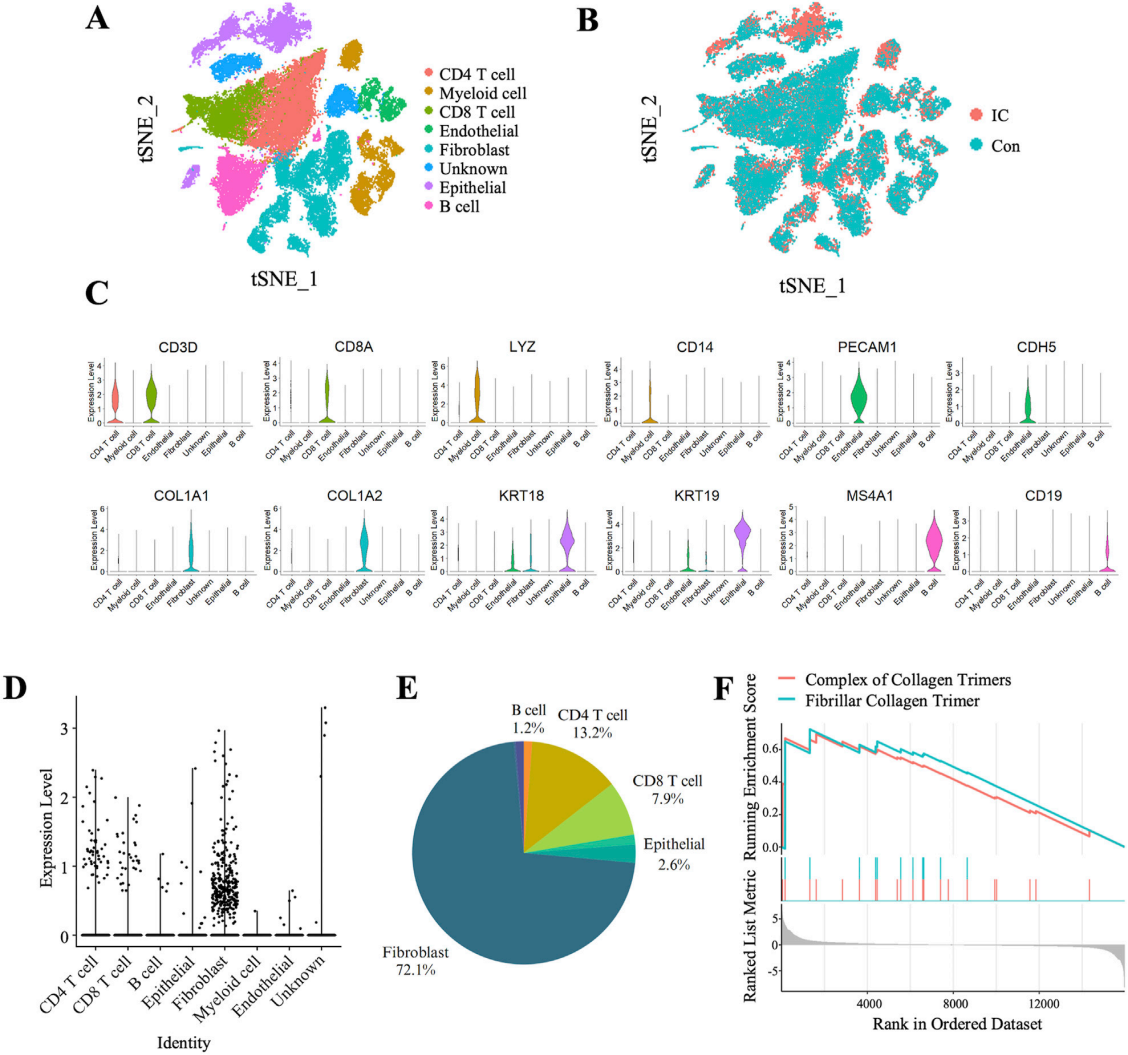
Predominant Expression of TRPC3 in Fibroblasts within Bladder Tissue and Upregulation of Fibrosis-Related pathways in CYP-Induced Cystitis rats. **(A, B)** t-SNE plot depicting cell cluster distributions within bladder tissue samples from two experimental groups. **(B)** Comparison of cell clusters between the two experimental groups. **(C)** Violin plots depicting the expression levels of selected marker genes across the identified clusters. **(D)** Violin plots demonstrating the predominant expression of TRPC3 in fibroblasts, **(E)** with fibroblasts constituting 72.1% of the TRPC3-expressing cells. **(F)** GSEA of pathways associated with fibrosis.

We analyzed single-cell RNA sequencing data from the GSE175526 dataset, which included bladder samples from five IC patients and two control patients. The distribution of cell populations across the sample groups was visualized using the t-SNE algorithm, as shown in Figures 4A, B. Seven distinct cell clusters were identified based on the expression of canonical markers, including CD4^+^ T cells (CD3D+/CD4+), CD8^+^ T cells (CD3D+/CD8A+), myeloid cells (CD14+/LYZ+), B cells (CD19+/MS4A1+), endothelial cells (CDH5+/PECAM1+), fibroblasts (COL1A1+/COL1A2+), and epithelial cells (KRT18+/KRT19+) (Figure 4C). Notably, TRPC3 expression was predominantly localized in fibroblasts, accounting for 72.1% of the total TRPC3 expression (Figures 4D, E). Further analysis using GSEA of bulk transcriptomic data revealed that fibrosis-related pathways were significantly upregulated in CYP-induced cystitis rats compared to controls (Figure 4F).

### Inhibiting TRPC3 with Pyr3 alleviated bladder fibrosis through TGF-β/smad pathway in CYP-Induced cystitis rats

**FIGURE 5:**
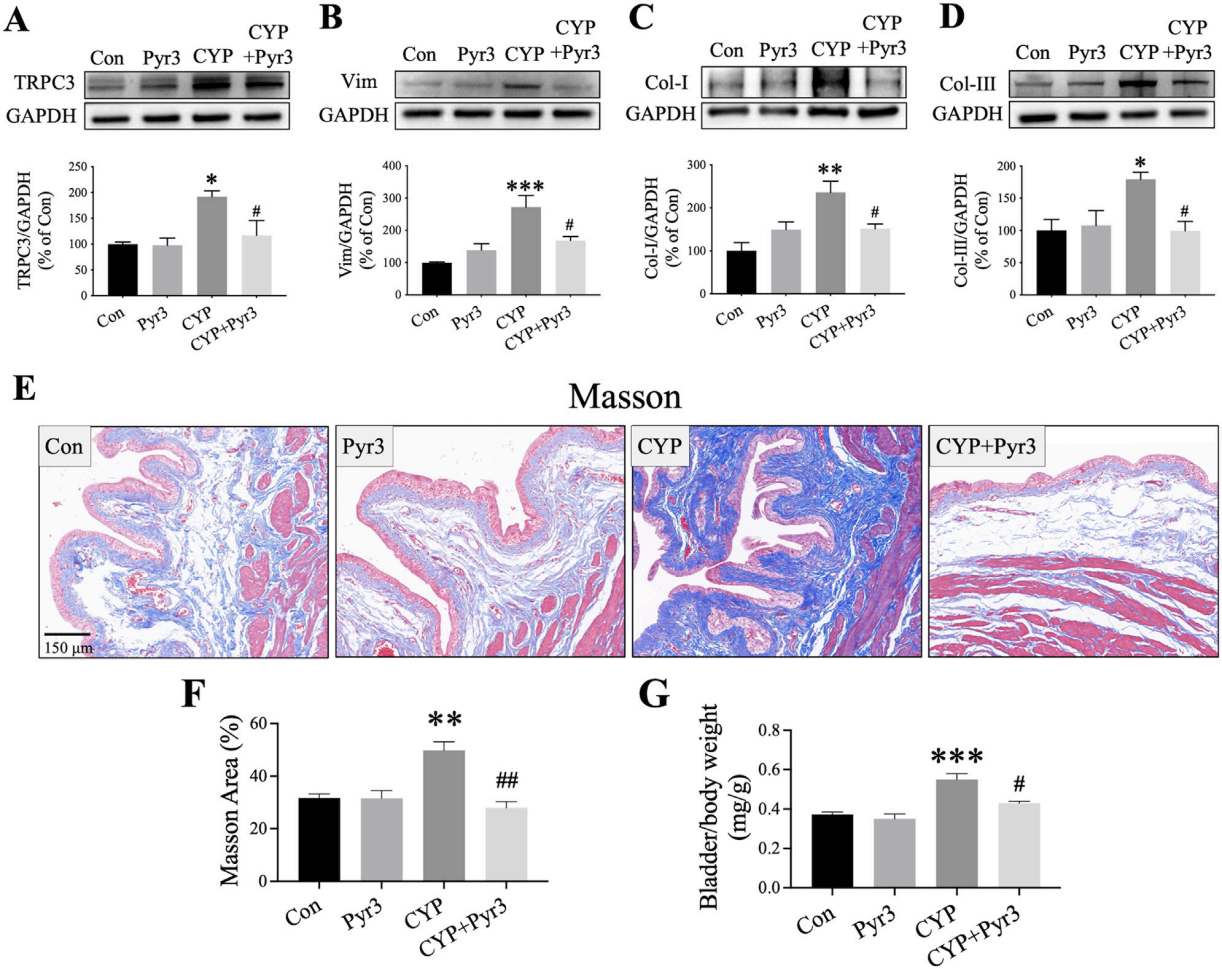
Pyr3 Alleviated Bladder Fibrosis in CYP-Induced Cystitis Rats. **(A–D)** Pyr3 treatment reversed the overexpression of fibrosis markers Vimentin (Vim), Collagen-I (Col-I), and Collagen-III (Col-III) in the bladders of CYP-induced cystitis rats. n = 4 per group. **(E)** Masson staining revealed that Pyr3 reduced collagen deposition in the bladder of CYP-induced cystitis rats. n = 4 per group. Statistical significance was determined using one-way ANOVA followed by the Tukey post hoc test: *P < 0.05, **P < 0.01 and ***P < 0.001 vs Con group, ^#^ *P* < 0.05 vs CYP group.

GSEA analysis of the bulk transcriptome and single-cell RNA sequencing revealed that TRPC3 may play a role in the development of fibrosis in CYP-induced cystitis. To test this hypothesis, the study further assessed the expression levels of fibrosis markers such as Vim, Col-I, and Col-III to evaluate bladder fibrosis across different experimental groups. Initial findings confirmed that Pyr3 effectively inhibits the overexpression of TRPC3 in the bladders of CYP-induced cystitis rats (*P* < 0.05, Figure 5A). As Figures 5B–D shown, the fibrosis markers Vim, Col-I, and Col-III are upregulated in the bladder of CYP-induced cystitis rats (*P* < 0.001, *P* < 0.01 and *P* < 0.05, respectively). However, Pyr3 significantly reversed the overexpression of these markers (all *P* < 0.05). Additionally, Masson staining was performed to assess collagen deposition in the bladder tissues. The results indicates that the increased collagen deposition observed in the bladders of CYP-induced cystitis rats was significantly mitigated by Pyr3 treatment (*P* < 0.01, Figures 5E, F). Additionally, Pyr3 treatment also reduced the elevated bladder/body weight ratio in CYP-induced cystitis rats (*P* < 0.05, Figure 5G).

**FIGURE 6:**
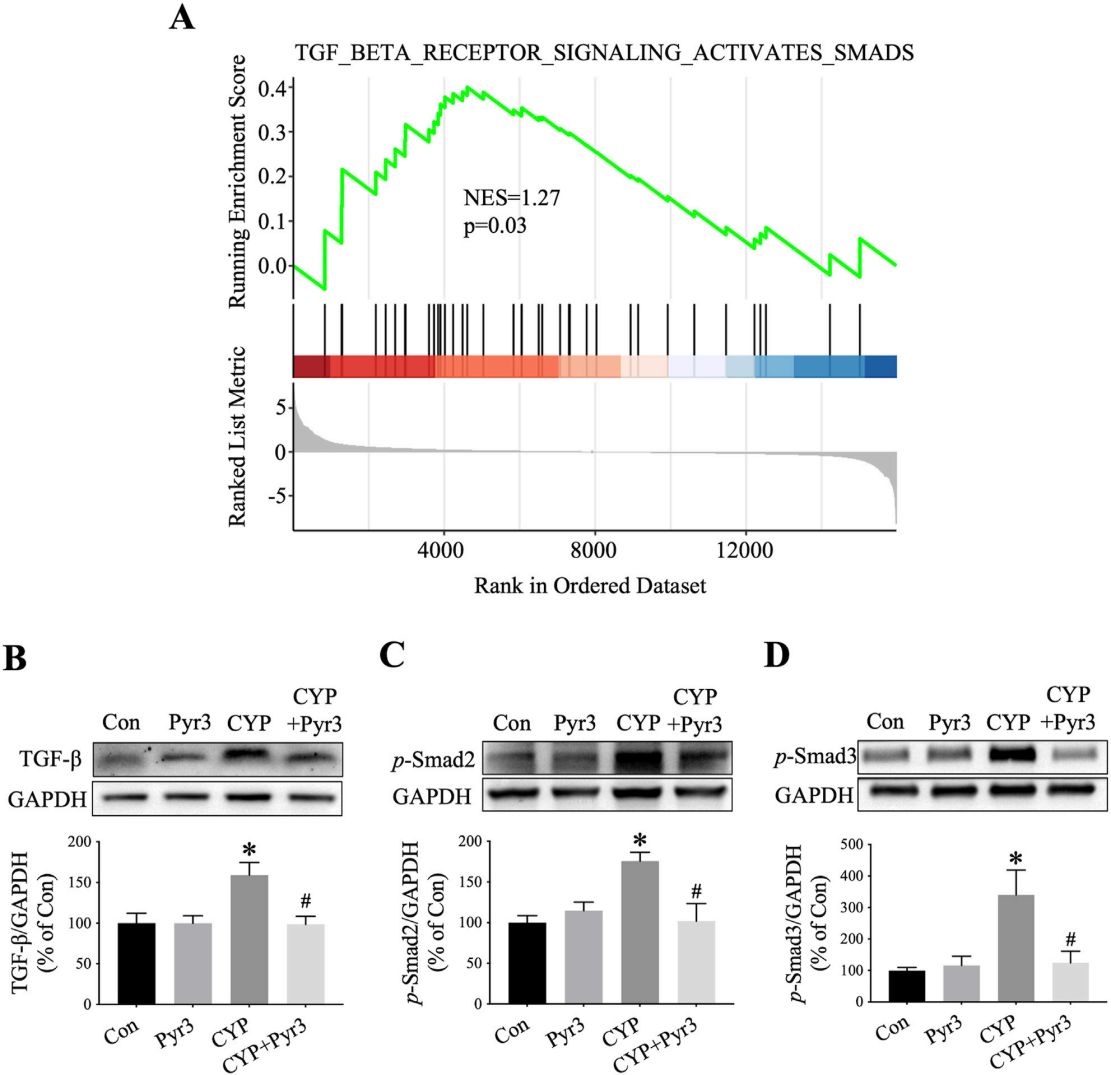
Pyr3 Alleviated Bladder Fibrosis Through TGF-β/Smad Pathway in CYP-Induced Cystitis Rats. **(A)** GSEA of the TGF-β/Smad pathway (B–D) Pyr3 reversed the upregulation of three key proteins of the TGF-β/Smad pathway—TGF-β, p-Smad2, and p-Smad3 in the bladder of CYP-induced cystitis rats. n = 3–4 per group. Statistical significance was determined using one-way ANOVA followed by the Tukey post hoc test: *P < 0.05 vs Con group, ^#^ *P* < 0.05 vs CYP group.

TGF-β is a pivotal fibrogenic cytokine that initiates a signaling cascade, leading to the activation of Smad proteins. Once activated, these Smad proteins translocate to the nucleus and activate genes associated with fibrogenesis (Meng et al., 2016). The role of the TGF-β/Smad signaling pathway in bladder fibrosis, particularly in conditions like IC/BPS, is well-documented (Shih et al., 2021). In our study, GSEA analysis of the bulk transcriptome indicated a significant upregulation of the TGF-β/Smad pathway in CYP-induced cystitis rats compared to the controls (Figure 6A). Given the TGF-β/Smad pathway’s crucial involvement in fibrosis, especially bladder fibrosis, we examined its expression in the bladders of CYP-induced cystitis rats with and without Pyr3 treatment. As shown in Figures 6B–D, three key proteins of the TGF-β/Smad pathway—TGF-β, *p*-Smad2, and *p*-Smad3—are upregulated in the bladders of CYP-induced cystitis rats (all *P* < 0.05). Interestingly, treatment with Pyr3 reversed these upregulations (all *P* < 0.05). These findings suggest that TRPC3 modulated bladder fibrosis in CYP-induced cystitis rats via TGF-β/Smad pathway.

## Discussion

Our study found that both mRNA and protein expression levels of TRPC3 are upregulated in the bladder of CYP-induced cystitis rats. Treatment with Pyr3, a selective TRPC3 inhibitor, significantly alleviated suprapubic mechanical allodynia and bladder overactivity in these rats. Further analysis suggested that TRPC3 may modulate bladder fibrosis in CYP-induced cystitis through TGF-β/Smad pathway. These findings highlight TRPC3 as a potential therapeutic target for IC/BPS in clinical practice, particularly in addressing the development of bladder fibrosis.

Bladder fibrosis plays a critical role in the progression of IC/BPS. This fibrosis can result from chronic inflammation due to persistent irritation as urine penetrates an impaired bladder epithelial barrier. Patients with severe fibrosis typically experience significantly higher urinary frequency and reduced bladder capacity compared to those with moderate or mild fibrosis (Kim et al., 2017). Additionally, IC/BPS patients who are unresponsive to conservative treatments—such as behavioral modifications, dietary changes, medications, and hydrodistension—often have severe bladder fibrosis (Clemens et al., 2022a). Currently, effective treatment options for refractory IC/BPS, particularly in cases involving an end-stage small fibrotic bladder, are limited (Clemens et al., 2022b). Recently, several potent antifibrotic agents including tocotrienols (Iguchi et al., 2023), pirfenidone (Ko et al., 2024), and N-acetylcysteine (Ryu et al., 2019) have demonstrated efficacy against bladder fibrosis across diverse animal models. However, clinical trials evaluating antifibrotic agents remain exceedingly limited. Furthermore, patients with refractory IC/BPS, particularly those presenting with end-stage small fibrotic bladders, have been significantly underrepresented in clinical trial populations. For these individuals, major surgeries such as supratrigonal cystectomy with augmentation cystoplasty may be necessary despite the presence of severe and unmanageable complications (Mateu Arrom et al., 2019; Queissert et al., 2022). Recognizing that bladder fibrosis is a key mechanism underlying refractory IC/BPS in patients with an end-stage small bladder, we investigated the mechanisms of bladder fibrosis to identify new therapeutic targets. Multiple studies have highlighted the involvement of TRPC channels, including TRPC1, TRPC3, and TRPC6, in cardiac and renal fibrosis (He et al., 2014; Camacho Londoño et al., 2015; Saliba et al., 2015; Wu et al., 2017; Saliba et al., 2019; Wang et al., 2024). We hypothesized that targeting TRPC channels could be a breakthrough strategy for addressing bladder fibrosis in IC/BPS. Our findings from bulk RNA sequencing and single-cell RNA sequencing analyses of bladder tissue indicated a significant role for TRPC3 in bladder fibrosis associated with IC/BPS. This role was further confirmed by improved bladder pain and overactivity following selective inhibition of TRPC3. In conclusion, TRPC3 appears to be a promising therapeutic target for reversing bladder fibrosis and alleviating bladder pain and overactivity in IC/BPS. These findings provide a strong rationale for developing TRPC3-targeted therapeutic agents for future clinical trials. Additionally, we propose further investigation into the pathogenic role of TRPC3 in other high-prevalence urological complications, including diabetes-associated bladder fibrosis, bladder outlet obstruction-induced fibrotic remodeling, and ketamine-induced bladder fibrosis.

The mechanism underlying bladder fibrosis in IC/BPS are complex and multifaceted. Inflammation is widely recognized as a crucial factor contributing to bladder fibrosis. For instance, treatments like curcumin (Shih et al., 2021), emodin (Lai et al., 2023), bladder wall injections of mesenchymal stem cells (Furuta et al., 2018), and downregulation of microRNA-132 (Song et al., 2019) have shown promise in reducing bladder fibrosis and improving urinary symptoms in LPS/PS-induced IC/BPS model by inhibiting bladder inflammation. Additionally, signaling pathways such as JAK-STAT (Song et al., 2019; Hou et al., 2021; Lai et al., 2023) and TGF-β/Smad (Shih et al., 2021; Lai et al., 2023) have been identified as significant contributors to bladder fibrosis in this context. Our study also identified the overexpression of various markers of bladder fibrosis, including Vim, Col-I, and Col-III, along with activation of the TGF-β/Smad pathway, in the bladder tissue of CYP-induced cystitis rats. We found that TRPC3 may modulate bladder fibrosis in IC/BPS through TGF-β/Smad pathway.

However, other mechanisms by which TRPC3 influences bladder fibrosis in IC/BPS require further investigation. For example, calcium influx via TRPC3 can affect mTOR signaling (Morales et al., 2007), potentially leading to the progression of bladder fibrosis (Qiao et al., 2018). Additionally, the regulation of calcium ion homeostasis by TRPC channels is essential for proper smooth muscle function. Research on rabbit models with partial bladder outlet obstruction has shown a significant disruption in sarcoplasmic reticulum function, which plays a crucial role in maintaining calcium ion balance. This disruption was found to be closely linked to bladder decompensation and reduced contractility (Stein et al., 2001). The above findings suggest that TRPC3 may directly or indirectly influence the development of bladder fibrosis. Building upon the established paradigm that TGF-β signaling (Evans et al., 2003) and calcium homeostasis (Castella et al., 2010) act as master regulators of fibroblast phenotypic transition, we hypothesize that TRPC3-mediated calcium dyshomeostasis synergizes with TGF-β/Smad signaling to orchestrate fibroblast activation and subsequent differentiation into myofibroblasts. These activated fibroblasts likely secrete pro-inflammatory mediators, including ATP and IL-1β, which amplify local inflammatory responses, exacerbate tissue damage, and directly contribute to detrusor overactivity (through ATP-induced smooth muscle contraction) and nociceptive sensitization (via IL-1β-mediated neuronal hyperexcitability). In addition to these mechanisms, increased release of ATP by the urothelium has been shown to enhance the contractile responses of the detrusor muscle, resulting in increased sensitivity in animal models of IC/BPS. Similar mechanisms may also be activated in a damaged and fibrotic urothelium that fails to perform its barrier function or maintain normal activity, which could potentially lead to detrusor overactivity (Ferguson et al., 2015; Avci et al., 2024) To address this mechanistic hypothesis, our future investigations will systematically dissect the spatiotemporal dynamics of TRPC3-dependent Ca^2+^ oscillations and their molecular crosstalk with TGF-β/Smad phosphorylation cascades within the fibrotic bladder microenvironment characteristic of IC/BPS.

Honestly, this study does have several limitations. First, it was conducted with a relatively small sample size, which may restrict the generalizability of the findings. Second, this study centered mainly on the classical TGF-β/Smad pathway associated with fibrosis. Other potential pathways and mechanisms involved in bladder fibrosis were not investigated, which could provide a more comprehensive understanding of the disease process. Third, the findings have not yet been validated in clinical trials involving human participants, which limits their immediate clinical applicability. These limitations underscore the necessity for further research with larger sample sizes, extended study durations, and a broader examination of other potential pathways and mechanisms. Moreover, confirming these findings in human clinical trials is crucial for facilitating their translation into clinical practice.

## Conclusion

TRPC3 activation plays a crucial role in the bladder fibrosis associated with IC/BPS. Pyr3 has been shown to reduce suprapubic mechanical allodynia and bladder overactivity in IC/BPS by inhibiting TGF-β/Smad pathway. Thus, targeting TRPC3 inhibition may present a promising therapeutic approach for IC/BPS in clinical practice, especially in addressing bladder fibrosis.

## Data Availability

The data presented in this study are deposited in the GEO repository, accession number GSE293339.
